# Attitudes towards Tourette syndrome and schizophrenia amongst psychology students

**DOI:** 10.3389/fpsyg.2026.1894805

**Published:** 2026-09-07

**Authors:** Catharina Cramer, Anne Kleine Büning, Simon Schmitt, Kirsten Müller-Vahl, Natasha Wood, Valerie Brandt

**Affiliations:** 1 Department of Psychiatry, Social Psychiatry and Psychotherapy, Hannover Medical School, Hannover, Germany; 2 Department of Psychiatry, Psychotherapy and Psychosomatics, RWTH Aachen University, Aachen, Germany; 3 Institute for Translational Neuroscience and Clinical Psychology, RWTH Aachen, Aachen, Germany; 4 School of Psychology, Center for Innovation in Mental health, University of Southampton, Southampton, United Kingdom

**Keywords:** media, psychology students, schizophrenia, stereotypes, stigma, Tourette syndrome

## Abstract

**Introduction:**

Both people with schizophrenia (SCZ) and Tourette syndrome (TS) have been found to be commonly stigmatized, but stereotypes held for both have never been directly compared. Clinicians and students of healthcare professions have been shown to also be affected by stereotypical beliefs. Media portrayals frequently perpetuate stereotypes, while accurate depictions could raise awareness and reduce stigma.

**Methods:**

The aim of this study was to compare stereotypes regarding SCZ and TS and their associations with social media consumption. We conducted an online survey amongst psychology students in the UK (*N* = 69, age *M* = 20.48 ± 1.75, range 18–26 years, 57 females).

**Results:**

Overall, both diagnoses were seen neutrally. Participants felt neutral about or disagreed with most stereotypes. However, SCZ was perceived as significantly unpredictable. In comparison to SCZ, people with TS were seen as less dangerous, violent, disruptive and unpredictable. Correlations did not show significant associations between daily hours spent on social media, seen portrayals, the belief that the media influences subjective beliefs, and negative attitudes towards TS/SCZ.

**Discussion:**

Our results suggest neutral to positive attitudes towards SCZ and TS in current psychology students. Compared to TS, SCZ is more affected by stereotypes. Given the small, local (UK) sample in this study, future studies should include more large-scale global samples to elicit a potential shift in attitudes towards mental health conditions in young healthcare professionals and its relation to the media.

## 1 Introduction

Both schizophrenia (SCZ) and Tourette syndrome (TS) are chronic psychiatric diagnoses. Their global prevalences are estimated 0.62% (95% confidence interval (CI): 0.51%−0.76%) for SCZ (Zhang et al., 2026) and 0.5% (95% CI: 0.3%−0.8%) for TS (Jafari et al., 2022).

According to DSM-5, TS is characterized by multiple motor and at least one vocal tic, persisting for at least 1 year, with an onset before age 18 (American Psychological Association, 2013). However, first tics typically manifest in early childhood at age 4 to 6 years (Bloch and Leckman, 2009). Although relatively rare in TS (Nilles et al., 2023, 2025), complex tics, such as coprolalia (involuntary swearing) and copropraxia (involuntary obscene gestures), are frequently presented on TV and social media channels, and described as being typical of the diagnosis (Calder-Sprackman et al., 2014; Zea Vera et al., 2022).

As per DSM-5 criteria, SCZ is characterized by at least two main symptom categories: Positive symptoms, such as delusions, hallucinations, disorganized speech, and less commonly disorganized or catatonic behaviors, as well as negative symptoms, including avolition, alogia, anhedonia, and blunted affect. These symptoms must persist for at least 1 month. However, such first psychotic episodes with positive symptoms are typically preceded by a longer prodromal period with general disturbances and negative symptoms, which must be present for at least 6 months and must cause functional impairment. First symptoms of SCZ typically occur in early adulthood with a median onset age of 25 years (Solmi et al., 2022).

Stigmatization is considered a complex social process according to recent definitions (Link and Phelan, 2006; Pescosolido and Martin, 2015; Goffman, 2022). Link and Phelan introduced the following conceptualization: stigma starts with labeling of interpersonal differences followed by subsequent linkage to negative or undesirable attributions, so-called stereotypes. Holding stereotyped labels can cause social separation into distinct groups of people without (‘us') and with the label (‘them'), often along with status loss and discrimination of the labeled group (Link and Phelan, 2001).

Both TS and SCZ are psychiatric diagnoses that have been surrounded by misconceptions, societal stereotypes, and stigma for a long time. Despite advances in mental health awareness and advocacy efforts, negative attitudes towards these diagnoses persist in most societies (Holtz and Tessman, 2007; Hori et al., 2011; Park et al., 2018; Cox et al., 2019). Importantly, stigma is associated with reduced help-seeking, which may result in a higher risk of maintaining symptoms (Doll et al., 2021). Both diagnoses are linked to alterations in dopaminergic circuits, and recommended treatments are antipsychotic medication (McCutcheon et al., 2020, 2025; Müller-Vahl et al., 2022). Treatments require treatment adherence, which can be hindered by stigma amongst healthcare professionals or internalized self-stigma (Buchman-Wildbaum et al., 2020; Doll et al., 2021; Chen et al., 2023; Pring et al., 2025), underlining the importance of investigating stereotypes regarding these two diagnoses.

The diagnosis of SCZ causes a significant, recently increasing burden, especially in young individuals (Solmi et al., 2023). In contrast to major depressive disorder, there was no decline in stigma against people with SCZ over the past decade (Reisinger and Gleaves, 2023; Schomerus et al., 2023). Likewise, many individuals with TS describe reduced quality of life, mainly driven by comorbidities including obsessive-compulsive disorder, attention-deficit/hyperactivity disorder, and depression (Huisman-van Dijk et al., 2019), but also by tics, pain due to the tics, internalized self-stigma, social exclusion, and bullying (Pring et al., 2025). Young people with TS have difficulties with peer acceptance and even healthcare professionals, such as clinicians, hold incorrect beliefs about the origin and controllability of tics (Marcks et al., 2004; Cox et al., 2019).

The importance of awareness and knowledge of TS for school psychologists and teachers has also been emphasized to support affected children with TS associated difficulties (Wodrich, 1998; Glassman, 2010; Ludlow et al., 2022). Missing knowledge and false ideas about TS have been shown in interviews amongst eight UK teachers (Ludlow et al., 2022), who reported to have retrieved information from TV shows. A survey comparing attitudes towards epilepsy and TS within one UK medical school showed that the included students were less likely to know someone with TS personally, but did more frequently report to know a famous person with TS (e.g., footballer, reality TV star) than with epilepsy (Cara Katona, 2013). Approximately 5% of the healthcare students even responded they would not like their children to play with someone with TS (Cara Katona, 2013).

One factor that may contribute to the formation and perpetuation of stereotypes is the (social) media portrayal of SCZ and TS (Owen, 2012; Calder-Sprackman et al., 2014; Pring et al., 2025). Many societies hold misleading ideas of SCZ as ‘split personalities', substantially driven by media portrayals (Schomerus et al., 2007; Böge et al., 2025). This can be traced back to metaphorical and devaluating usage of the term in books in the 20th century, continuing until today in different well-known international newspapers (Duckworth et al., 2003; Hoffmann-Richter et al., 2003; Borsche et al., 2007). With regard to visual media, a study including 41 movies displaying at least one character with SCZ revealed a bias towards showing positive symptoms as well as violent, suicidal, and homicidal behaviors (Owen, 2012). Likewise, out of 35 YouTube videos (out of 4,200 screened) allegedly showing individuals with SCZ, only 12 actually represented typical symptomatology according to psychiatrists' ratings (Nour et al., 2017).

In 2014, Calder-Sprackman and colleagues analyzed 37 movies and television shows ostensibly depicting TS. The clips overrepresented coprophenomena and mostly included negative portrayals, suggesting that individuals with TS and their social surrounding experienced high levels of suffering due to tics (Calder-Sprackman et al., 2014). Of the 20 most popular TS YouTube videos that were uploaded until 2010 the majority showed negative, derogatory portrayals as well as parodic or satirizing depictions, mostly focusing on complex coprolalia (Fat et al., 2012). Beyond that, negative YouTube portrayals were associated with even more view counts and comments than positive and empathetic ones (Fat et al., 2012). Of the videos that were advertised as TS-related on the social media platform TikTok, most contained coprophenomena and self-injurious or aggressive behaviors (Zea Vera et al., 2022). Thus, these portrayals are not only inaccurate for TS, but are more typical of functional movement disorders (FMD) and may promote their spread (Zea Vera et al., 2022).

To counter these misperceptions, several Eastern Asian countries changed the term SCZ to different terminology, e.g., ‘attunement disorder' in Korea, yielding partly positive effects on its public perception (Chiu et al., 2010; Yamaguchi et al., 2017; Kim et al., 2023). In Europe, German clinicians strongly recommended a similar adaptation recently (Böge et al., 2025). Moreover, a broad American survey empirically underpinned the stigma-reducing potential of a renaming (Mesholam-Gately et al., 2021; Böge et al., 2025). Similarly in TS, renaming is currently being researched and discussed to reduce stigma (Raffaele et al., 2025). However, the authors highlighted the importance of reattributing associated stereotypes alongside renaming, because SCZ is often connected to personality traits or behaviors like ‘dangerousness', ‘violence', or being ‘unpredictable' (Böge et al., 2025), which are present not only among lay people, but also in mental health professionals (Valery and Prouteau, 2020; Reisinger and Gleaves, 2023). However, a quasi-randomized controlled study from Italy showed that an educational training was effective in reducing psychology students' stereotypes about SCZ and to focus on psychosocial causes and treatment effectiveness instead (Magliano et al., 2016). Psychology student's attitudes towards psychiatric diagnoses are of particular importance as they can influence affected peoples' opportunities of finding a psychologist for treatment. A recent systematic review of mental health stigma across various healthcare disciplines found evidence supporting the hypothesis of lower stigma levels in professionals with personal patient contacts.

Taking the medias' influence over public opinion into account, inaccurate media portrayals have been described as part of structural stigmatization (Pring et al., 2025). Therefore, its relation to individual stereotypes should be further investigated (Hatzenbuehler and Link, 2014; Pring et al., 2025). The current study compares stereotypes about TS and SCZ directly because they share some stereotypes that they do not necessarily share with other common diagnoses, such as major depression or anxiety disorders. For instance, the assumption that people with TS/SCZ may behave in an erratic or unpredictable manner (Magliano et al., 2016; Malli and Forrester-Jones, 2017; Böge et al., 2025), and might visibly disrupt social norms in public spaces (Mannarini et al., 2022; Pring et al., 2025). In this work, our aim was therefore (i) to analyze whether TS and SCZ are linked to undesirable attributes such as ‘dangerousness', ‘disruptiveness', ‘violence' and being ‘unpredictable' by psychology students (ii) to compare participants' agreements with stereotypes related to TS and SCZ, (iii) to test, if people hold fewer stereotypical beliefs when personally knowing someone with TS or SCZ, respectively, and (iv) to investigate the relationship between media usage, belief in media's influential potential, and attitudes towards TS and SCZ, shedding light on how people perceive the potential influence of media representations on public perceptions of these diagnoses.

## 2 Methods

### 2.1 Participants

**Figure 1:**
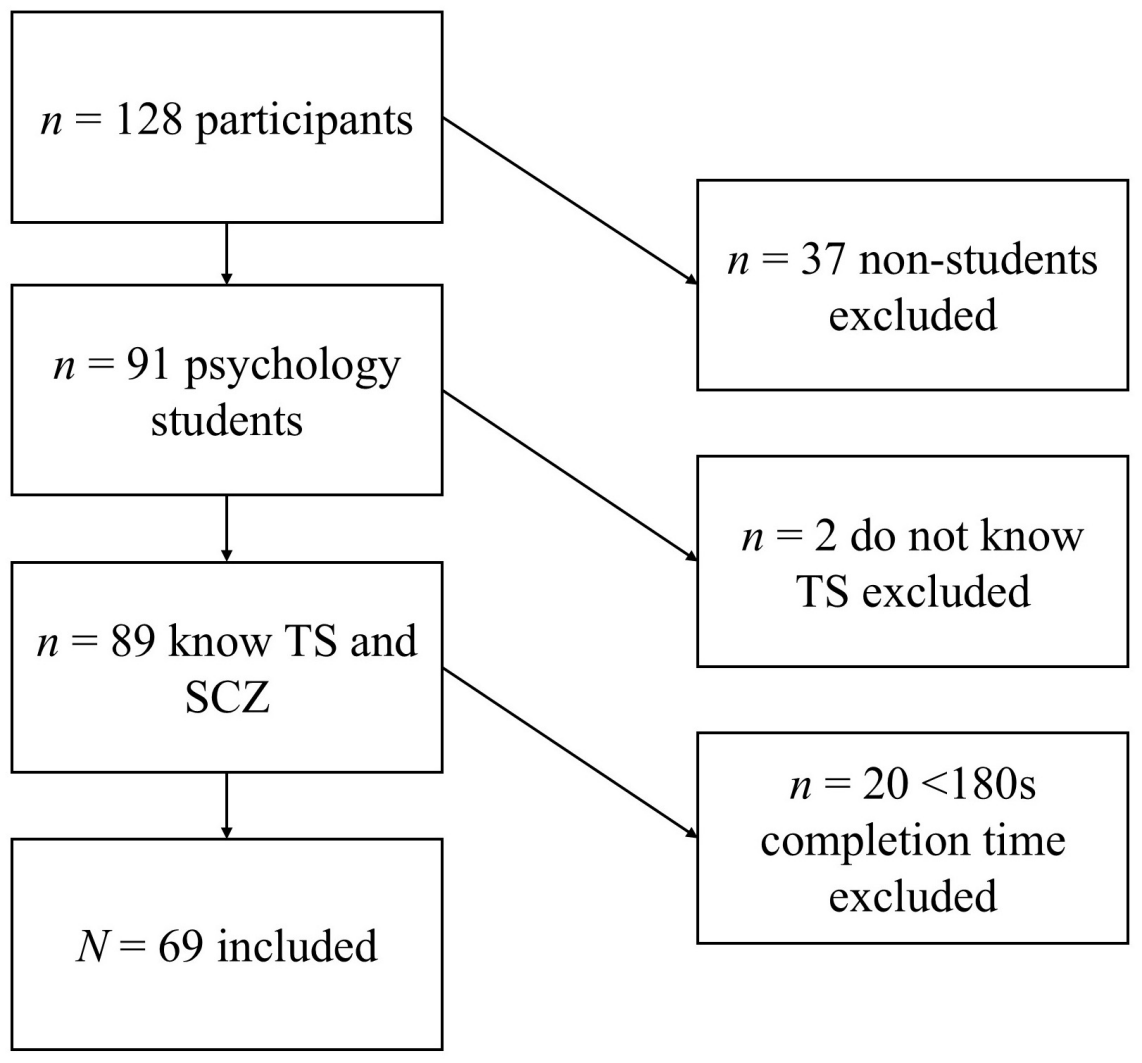
Flow chart of participant inclusion.

Participants were recruited from the student pool at University of Southampton and through social media channels from March to April 2024. Those enlisted through the university software Sona Systems (https://www.sona-systems.com) received an incentive of three research credits for taking part, whereas those recruited through social media channels received no incentive. The Southampton Ethics Committee approved this study according to the Declaration of Helsinki (ERGO number 91940). *N* = 128 participants filled out the online survey after providing informed consent and confirming they were ≥18 years old. Demographic information requested included age, gender, ethnicity, and highest educational level. Non-students (*n* = 37, 28.9% of *n* = 128) were excluded during the inclusion process for data analysis (Figure 1). Students not knowing TS (*n* = 2, 2.2% out of *n* = 91 students) or SCZ (*n* = 0), or took under 3 min (*n* = 20, 21.98% of *n* = 91 students) to complete the survey were excluded. We included *N* = 69 out of *n* = 91 participating psychology students in our analysis. Participants were aged on average *M* = 20.48 years (*SD* = 1.75, range 18–26 years), *n* = 11 (15.94%) identified as male, *n* = 57 (82.61%) as female and *n* = 1 (1.45%) preferred not to say. Regarding education, *n* = 1 (1.45%) held GCES or equivalent, *n* = 50 (72.46%) A-levels or equivalent, and *n* = 18 (26.09%) a university degree or equivalent. In our sample *n* = 62 (89.86%) were of White ethnicity, *n* = 4 (5.8%) Asian, *n* = 4 (5.8%) Mixed, and *n* = 1 (1.45%) Arab. Participants spent on average *M* = 5:53 min to fill out the questions (*SD* = 4:15 min, range 3:06 min−34:27 min). We did not find consistent effect sizes in previous literature and therefore conducted a power analysis using G^*^Power for paired *t*-tests, assuming small to medium effect size Cohen's *d* = 0.4), setting α = 0.05 and power (1- β) = 0.95 which determined a target sample size of *n* = 70 participants (Faul et al., 2007).

### 2.2 Design and materials

Participants had to answer an anonymous survey with 24 multiple-choice questions on Qualtrics (Provo, UT). To determine media usage, participants were asked how many hours they spend on social media daily (0 to 24 h). We further assessed the frequency of media portrayals seen in the media for each diagnosis, such as characters in films and TV programs, social media posts, or similar on a 4-point Likert scale (0 = ‘never' to 3 = ‘quite often'). The literature was searched for common stereotypes about TS and SCZ, and each stereotype was turned into an item, so participants could rate them on a 5-point Likert scale (0 = ‘strongly disagree' to 4 = ‘strongly agree'). In addition, one TS specific question was included: ‘Individuals with TS always swear'. The set of statements included general attributes: ‘I view people with SCZ/TS as ‘dangerous/ disruptive/ unpredictable/ violent/ negatively'. The items containing stereotypical statements available for both diagnoses were summed up into one negative-attitude-score for each diagnosis in order to use them in Pearson correlations (possible range: 0 to 24 for SCZ/TS each). We calculated Cronbach's α as a reliability measure of the internal consistency of these scores, which was good for SCZ (Cronbach's α = 0.83) and acceptable for TS (Cronbach's α = 0.73).

We further asked participants to indicate their overall impression of each diagnosis on a 5-point Likert scale (‘very negative' to ‘very positive'). Furthermore, participants were asked how often they had come across negative representations of SCZ/TS in the media, and whether participants had the impression that their individual attitudes towards the psychiatric diagnoses were influenced by the media. These were assessed with the same 5-point Likert scale as above. Moreover, we asked participants if they had ever had contact with someone with TS or SCZ. Finally, participants were asked if they would willingly associate with people with TS/SCZ (‘I would not associate willingly with those with SCZ/TS').

### 2.3 Statistical analyses

(i) Two tailed one-sample *t*-tests were applied to assess whether our sample agreed with stereotypical statements on people with SCZ/TS (reference number 2 = ‘neither agree nor disagree'), and to measure their overall impression (‘very negative' to ‘very positive', reference number 2 = ‘neutral'). The threshold for significance was Bonferroni-Holm adjusted per SCZ/TS.

(ii) Two tailed paired *t*-tests were utilized to test for differences in stereotypical beliefs (‘dangerous', ‘disruptive', ‘violent', ‘unpredictable', ‘not associate with'), impression of media depictions (‘negative portrayals', ‘media influences opinion'), negative-attitude scores and overall impressions between the two diagnoses. Bonferroni-Holm corrections were applied to correct for multiple testing of stereotypes for multiple paired *t*-tests per SCZ/TS separately. Cohen's *d* was calculated as a measure of effect size for all statistically significant *t*-test results.

(iii) Two tailed independent sample *t*-tests were used to test whether negative attitude scores differed between participants who reported to know someone with TS/SCZ or not with a threshold for significance of α =0.05. The assumption of normality for the paired *t*-tests, and normality of the variables for one-sample *t*-tests was inspected using Shapiro-Wilk tests and was visually inspected with histograms. The TS and SCZ negative-attitude scores and their difference were normally distributed, all other stereotypes and their differences (SCZ vs. TS) were not. Although deviations were found, the *t*-tests were considered appropriate because of their robustness in samples with *n* > 30 (Lumley et al., 2002).

(iv) Pearson correlations were applied to investigate the relationship of hours of social media consumption, how often participants came across portrayals of SCZ/TS, the belief in media's influence over opinions on negative-attitude scores. The threshold for significance was set to α = 0.05. The assumption of linearity was inspected visually using scatterplots. The assumption of normality was tested using Shapiro-Wilk and was fulfilled for the negative-attitude-scores, but was violated for the other included variables which was tolerated given the methods robustness to moderate departures (Zeller and Levine, 1974). *P*-values were Bonferroni-Holm corrected.

## 3 Results

### 3.1 Stereotypes about Tourette syndrome and schizophrenia

(i) On average, participants rated their overall impression of SCZ as neutral (*M* = 2.18, *SD* = 0.86, *t* = 1.72, *p*_(*Holm*)_ = 0.180) and for TS as slightly and significantly positive (*M* = 2.58, *SD* = 0.77, *t* = 6.11, *p*_(*Holm*)_ < 0.001).

One-sample *t*-tests (corresponding *M* and *SD* displayed in Table 1) showed that people with SCZ were significantly not viewed as dangerous (*t* = −3.66, *p*_(*Holm*)_ = 0.003, *d* = 1.03), or violent (*t* = −3.29, *p*_(*Holm*)_ = *0.0*06, *d* = 0.99), but as significantly unpredictable (*t* = 5.55, *p*_(*Holm*)_ < 0.001, *d* = 0.85). Participants would significantly willingly associate with a person with SCZ (*t* = −7.27, *p*_(*Holm*)_ < 0.001, *d* = 1.08) and disagreed significantly to associate SCZ with disruptiveness (*t* = −3.09, *p*_(*Holm*)_ = 0.009, *d* = 0.94). Participants significantly thought that SCZ was portrayed negatively in the social media (*t* = 12.31, *p*_(*Holm*)_ < 0.001, *d* = 0.72), but they were neutral about the media influencing their opinion (*t* = 0.34, *p*_(*Holm*)_ = 0.738).

**Table 1:** Paired *t*-test results of psychology students' agreement with stereotypical statements about Tourette syndrome and Schizophrenia.

Item	SCZ M (SD)	TS M (SD)	*T*	*p*	*d*
Overall impression	2.18 (0.86)	2.58 (0.77)	−4.20^***^	< 0.001	−0.76
Dangerous	1.54 (1.02)	0.49 (0.61)	8.63^***^	< 0.001	1.00
Disruptive	1.65 (0.94)	2.03 (1.13)	−2.72^**^	0.008	1.16
Violent	1.60 (0.99)	0.66 (0.68)	7.72^***^	< 0.001	1.00
Unpredictable	2.57 (0.85)	2.16 (1.00)	3.14^***^	< 0.001	1.08
Not associate with	1.04 (1.08)	0.39 (0.60)	4.84^***^	< 0.001	1.10
Negative media	3.07 (0.72)	2.25 (1.04)	6.14^*^	0.017	1.10
Media–Opinion	2.04 (1.08)	1.84 (1.18)	1.28	0.204	–

TS, Tourette syndrome; SCZ, schizophrenia; ‘Negative Media', frequency of encountering negative representations in the media; ‘Media–Opinion', extent that participants believe their opinions were influenced by the media. Agreement rating scale: 0 – ‘strongly disagree' to 4 – ‘strongly agree'.

Results of paired *t*-tests with *T*, Bonferroni-Holm corrected *p*-values (^*^p < 0.05, ^**^p < 0.01, ^***^p < 0.001) and Cohen's *d* for significant results.

One-sample *t*-tests showed that patients with TS were significantly not viewed as dangerous (*t* = −20.15, *p*_(*Holm*)_ < 0.001, *d* = 0.61) or violent (*t* = −16.17, *p*_(*Holm*)_ < 0.001, *d* = 0.68). They were rated neutrally when it came to disruptiveness (*t* = −0.21, *p*_(*Holm*)_ = 0.831) or being unpredictable (*t* = 1.33, *p*_(*Holm*)_ = 0.562). Participants were significantly likely to say that they would willingly associate with a person with TS (*t* = −21.96, *p*_(*Holm*)_ < 0.001, *d* = 0.6). They indicated that TS was portrayed neutrally in social media (*t* = 1.98, *p*_(*Holm*)_ = 0.208) and were neutral about the media influencing their opinion (*t* = −2.61, *p*_(*Holm*)_ = 0.524). One sample *t*-tests showed that our sample on average did significantly not associate TS with swearing (*M* = 1.27, *SD* = 1.09, *t* = −5.58, *p*_(*Holm*)_ < 0.001, *d* = 1.09). Descriptively, 19.1% agreed or strongly agreed that people with TS ‘always swear', 13.2% neither agreed nor disagreed and 67.7% disagreed or strongly disagreed with this statement (all item descriptives can be found in Supplementary Tables 1–3).

### 3.2 Comparison of stereotypes about Tourette syndrome and schizophrenia

**Figure 2:**
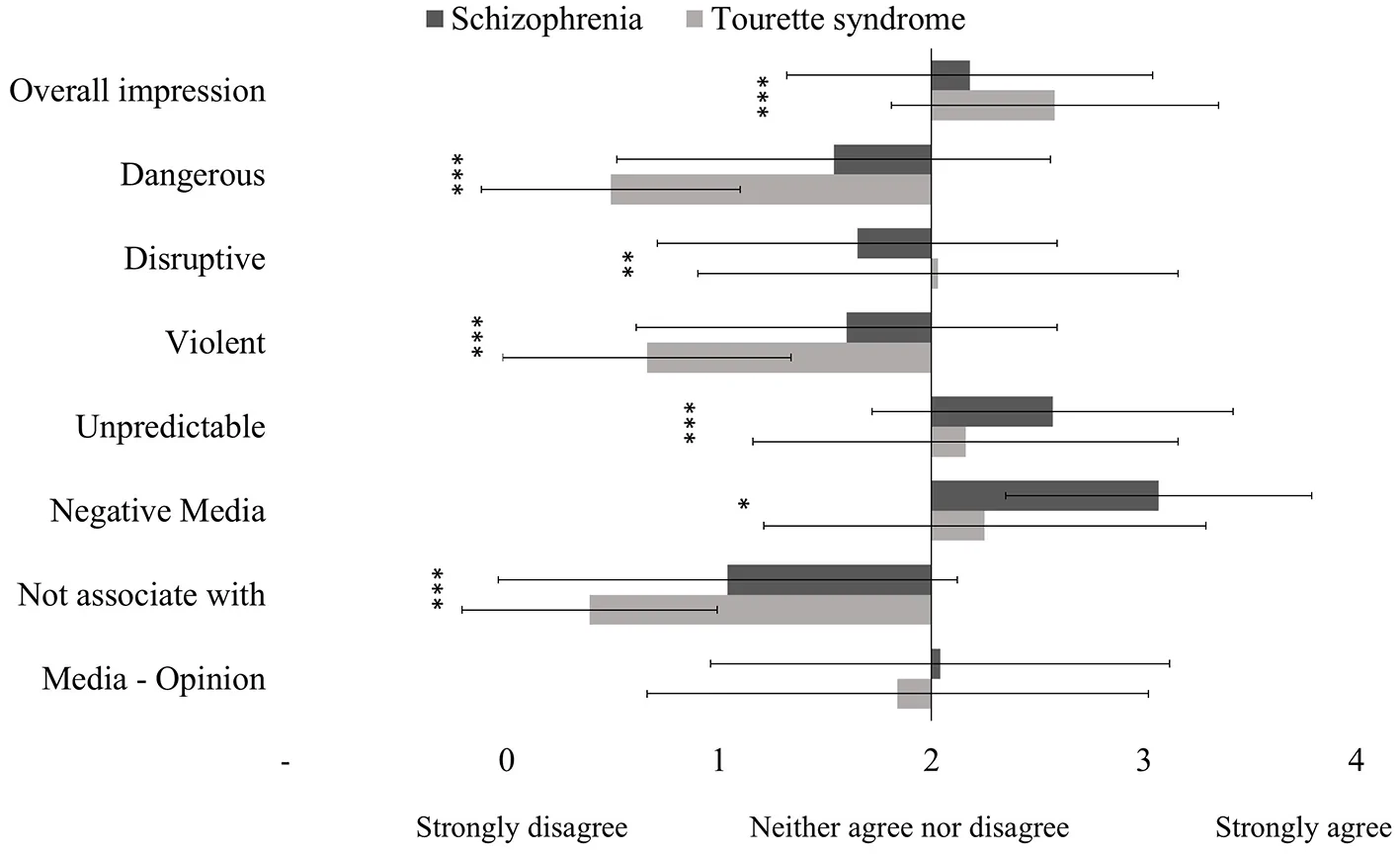
Agreement of psychology students with stereotypical statements regarding Tourette syndrome and schizophrenia.

(ii) When comparing TS and SCZ with paired *t*-tests (Table 1, Figure 2), we found the overall impression of TS was rated significantly more positive than for SCZ. People with SCZ were seen as significantly more dangerous, disruptive, violent, and unpredictable than people with TS. Our sample indicated to be significantly more willing to associate with someone with TS compared to SCZ. Moreover, our sample had the impression that SCZ was portrayed significantly more negatively in the media than TS. The belief that media influenced opinions did not differ significantly between SCZ and TS. The calculated negative attitude score was significantly higher for SCZ (*M* = 9.25, *SD* = 4.32, range 0–24) than for TS (*M* = 6.34, *SD* = 3.27, range 0–13, *t* = 5.62, *p* < 0.001, *d* = 4.23).

### 3.3 Social acquaintances and stereotypes

(iii) For TS, there were no significant differences in negative attitudes between subjects who described personal contact (*n* = 18, 26.1%, *M* = 6.44, *SD* = 3.38), compared to those who did not (*n* = 51, 73.9 %, *M* = 6.3, *SD* = 3.26, *t* = −0.16, *p* = 0.874).

Also, for SCZ negative attitude scores did not differ between subjects if personal contact was described (*n* = 9, 13%, *M* = 10.67, *SD* = 1.21) or not (*n* = 60, 87%, *M* = 9.03, *SD* =0.57, *t* = −1.06, *p* = 0.295).

### 3.4 Media and stereotypes

(iv) The students indicated to spend on average 4:09 h daily on social media (*SD* = 1:50 h, range 1–10 h). They reported they did not often come across media portrayals of people with SCZ (*M* = 1.41, *SD* = 0.69) or TS (*M* = 1.25, *SD* = 0.67, descriptives of this item in Supplementary Table 4). There were no significant correlations between daily hours spent on social media, the frequency with which participants came across SCZ/TS media portrayals, the belief that the media influenced subjective impression of SCZ/TS, and overall SCZ/TS attitude scores (Supplementary Tables 5, 6).

## 4 Discussion

In contrast to previous descriptions of negative attitudes towards TS and SCZ, our UK psychology student sample had neutral impressions of SCZ and positive attitudes towards TS (Valery and Prouteau, 2020; Böge et al., 2025; Pring et al., 2025). This subjective, general tendency of neutral to positive attitudes was confirmed in the detailed questions about stereotypical attributes, showing that people with SCZ were not viewed as dangerous, violent or disruptive. This pattern is atypical relative to broader previous literature (Angermeyer and Matschinger, 2004; Kruse and Dodell-Feder, 2025; Pring et al., 2025). However, people with SCZ were seen as slightly but significantly unpredictable, which has been reported to be a consistent stereotype (Read et al., 2006; Böge et al., 2025).

Our sample did not associate TS with any of these stereotypical attributes. TS was, on average, also not associated with swearing, which is a medially overrepresented TS stereotype (Fat et al., 2012; Olvera et al., 2021; Zea Vera et al., 2022). Recent studies described a rather low prevalence of 5% for coprolalia and 3% of copropraxia in children or adolescents with tic disorders (Nilles et al., 2023) and 8% coprolalia and 3% copropraxia in adults (Nilles et al., 2025), respectively. Our result is atypical of the pattern observed in previous studies, where TS was mostly associated with coprophenomena. Especially in healthcare professionals, this could promote misdiagnoses (Müller-Vahl et al., 2026). However, it should be noted that 19% of the sample still thought TS was associated with swearing.

When comparing TS and SCZ directly, people with SCZ were seen as significantly more dangerous, violent, disruptive, and unpredictable than people with TS. The data on actual violent and erratic behavior in people with SCZ is mixed. Even though a higher relative risk of violent perpetration, especially in young males with SCZ, in comparison to those without SCZ, was reported in a systematic review and meta-analysis, the authors highlighted that their findings were no proof of causal linkage (Whiting et al., 2022). Moreover, other contributing factors, e.g., economic disadvantages, have not been recorded systematically and are therefore understudied (Whiting et al., 2022). Our finding of relatively worse stereotypical impressions of SCZ compared to TS is in line with previous descriptions of SCZ being one of the most stigmatized psychiatric diagnoses (Valery and Prouteau, 2020). Participants also said they would rather associate with someone with TS than someone with SCZ. However, it is important to highlight that overall, both diagnoses have been rated mostly neutral to positively and the relatively worse opinions on SCZ do not reflect overall high stereotypes within our sample.

It should be noted that our sample was relatively small, however, only future psychologists were included in the survey. Moreover, our sample, consisting of psychology University students, was highly educated and already well-informed about psychiatric diagnoses. This likely contributed to the neutral to positive attitudes observed. Previously, psychologists have already been found to have less stigmatizing attitudes towards mental health disorders than other healthcare professionals (Kruse and Dodell-Feder, 2025). Given the stereotypes that still exist in professions such as psychology and medicine (Marcks et al., 2004; Chen et al., 2023), it is encouraging that our findings may point towards changing views of psychiatric diagnoses in specialists. However, these results are not generalizable to a broader population of young adults. One limitation of this study is the lack of a general public control group. Further limitations of generalizability are due to our sample's ethnic homogeneity (89.86% White) and gender imbalance with a majority of females (82.61%). While it should be noted that the sample was roughly representative of UK clinicians (in psychology), a more global approach to stereotypes towards mental health disorders would enhance our understanding in a boarder and more meaningful way. Gender differences regarding mental health were previously reported for Italian students with females being less stigmatizing than males (Pascucci et al., 2017). The same was true for Canadian, Spanish and Russian university students (Gallego et al., 2020), even though the differences were rather small. Non-significant gender differences of better knowledge about TS were found in female compared to male Saudi Arabian medical students and physicians (Alalwan et al., 2022). In contrast, another study found more female as compared to male medical and psychology students to perceive people with SCZ as dangerous (Fresán et al., 2018).

A study assessing stigmatizing vocabulary in newspaper articles from 1980 to 2018 indicated a steady, general stigma decline for physical, but not psychiatric diagnoses (Best and Arseniev-Koehler, 2023). The change in attitudes towards several physical diseases was interpreted to be a “cultural change” to more neutral/positive attitudes (Best and Arseniev-Koehler, 2023). The tendency to hold neutral to positive attitudes towards TS and SCZ in our pre-professional sample raises the question of whether a broad shift to more accepting and neutral attitudes is also starting to emerge for mental health conditions.

Attitudes in this sample did not differ between participants who had personal contact with either disorder, in comparison to those who did not. However, only quarter of our sample reported to have had personal contact with someone with TS and only almost a tenth with someone with SCZ, resulting in small numbers. Contact rates between the diagnoses noticeably differ, even though the diagnoses share a similar prevalence. Explanatory factors could be differences in the above described stereotypes, e.g. perceived dangerousness, which has been found to be linked to social distancing (Mannarini et al., 2022). However, even though we did not find any contact-dependent differences in attitude scores, a recent meta-analysis showed structured contact interventions to be more effective for stigma reduction in adults than theoretical education, whereas adolescents profited more from educational interventions (Corrigan et al., 2012). This could also explain why in our sample attitudes did not differ depending on personal contact, as we only asked for personal acquaintances and not structured patient contact, which was previously suggested to be beneficial in stigma reduction (Kruse and Dodell-Feder, 2025). Likewise, a systematic review found more positive attitudes towards SCZ in healthcare professionals who had lived experiences with affected persons (Chen et al., 2023). Another study found physicians and medical students to be more empathetic towards people with TS after completing educational patient-led TS presentations, highlighting the beneficial potential of including affected people's experiences into professional education (Graham et al., 2014).

In our sample, the amount of time spent on social media had no influence on attitudes. Participants did not often come across negative media depictions of both diagnoses, but they reported having seen more of SCZ than of TS. In future studies it would be interesting to see whether psychology students have seen more media content of psychiatric diagnoses than the general public, as higher interest in psychiatric diagnoses may influence personalized social media algorithms to show more related content. A systematic review of 12 studies addressing social media and stigmatization of psychiatric diagnoses found associations of positive posts with stigma reductions and vice versa (Ross et al., 2019). Apart from the valence of media portrayals (positive or negative), we would like to highlight the importance of their clinical accuracy. Misrepresentations may produce incorrect “knowledge”, not only in the general public, but also in non-specialized healthcare professionals and students in corresponding fields that are or will be responsible for diagnosis and treatment.

Counteracting stereotypes, enhancing communication, treatment-adherence, and well-being was successfully achieved in psychoeducational trainings for patients with SCZ and their relatives (Rodolico et al., 2022). This is not only important for people with TS/SCZ in their everyday life to prevent internalized self-stigma and exclusion, but also to improve the course of disease and well-being (Pring et al., 2025). People holding stereotypes may invest in aggressive, criticizing, and hostile interactions that are described as high expressed emotions (Peng et al., 2024). High expressed emotions in social surroundings of patients have been found to be a risk factor for worse SCZ outcome with early relapses, whereas warmth in interpersonal contact is a protective factor (Ma et al., 2021). The relevance of high expressed emotions on wellbeing has also been discussed for TS (Lee et al., 2020).

To further address this topic, more large-scale, global studies are needed to understand stereotypes, not only in pre-professionals, but also in healthcare professionals and the general public, as our sample has its limitations in generalizability. Moreover, updated studies about the valence and accuracy of media representations of TS/SCZ and experimental designs for directly measuring their influence on attitudes and openness to contact might be more revealing. Moreover, indirect measures of stereotypes might be used, as questionnaires are always subject to social desirability biases.

To summarize, this sample consisting of psychology students held neutral to positive attitudes towards TS and SCZ on average. When compared, attitudes towards TS were more positive than towards SCZ. While this may indicate a positive shift in attitudes towards individual mental health diagnoses, we would like to re-emphasize the importance of evidence-based, accurate, and empathetic media reports by patients, researchers, and clinicians. Furthermore, an integration of the discussion and interpretation of media representations of psychiatric disorders within the clinical and university context could be helpful. With rising interest in psychiatric diagnoses in the general public, especially on social media, we hope to enter a new era of broad stigma decline for psychiatric diseases.

## Data Availability

The raw data supporting the conclusions of this article will be made available by the authors, without undue reservation.
