# Supplementary material for: Attitudes towards Tourette syndrome and schizophrenia amongst psychology students
Source: Front Psychol. 2026 Sep 7;17:1894805. doi: 10.3389/fpsyg.2026.1894805 (PMC13590564; doi:10.3389/fpsyg.2026.1894805)
Supplement: Supplementary file 1 [file Supplementary_File_1.docx]

**Appendix**

**A1.**

|  | Percent (%) |
| --- | --- |
| 0 - Strongly disagree | 26.5 |
| 1 - Disagree | 41.2 |
| 2 - Neither agree nor disagree | 13.2 |
| 3 - Agree | 17.6 |
| 4 - Strongly agree | 1.5 |

*Agreement with the item ‚people with Tourette Syndrome always swear’ in percent (%).*

**A2.**

|  | Dangerous | Disruptive | Violent | Unpredictable | Would not associate with | Negative media representation | Media influenced my perception |
| --- | --- | --- | --- | --- | --- | --- | --- |
| 0 | 13.2 | 11.8 | 13.2 | 2.9 | 36.8 | 0 | 5.9 |
| 1 | 41.2 | 30.9 | 33.8 | 7.4 | 36.8 | 1.5 | 29.4 |
| 2 | 27.9 | 39.7 | 35.3 | 26.5 | 16.2 | 17.6 | 27.9 |
| 3 | 13.2 | 16.2 | 14.7 | 55.9 | 5.9 | 52.9 | 27.9 |
| 4 | 4.4 | 1.5 | 2.9 | 7.4 | 4.4 | 27.9 | 8.8 |

*Agreement with stereotypical statements regarding Schizophrenia in percent (%). ‘Strongly disagree’ = 0, ‘disagree’ =1, ‘neither agree nor disagree’= 2, ‘agree’ = 3, ‘strongly agree’ = 4.*

**A3.**

|  | Dangerous | Disruptive | Violent | Unpredictable | Would not associate with | Negative media representation | Media influenced my perception |
| --- | --- | --- | --- | --- | --- | --- | --- |
| 0 | 56.7 | 10.3 | 45.6 | 8.8 | 64.7 | 4.4 | 16.2 |
| 1 | 37.3 | 26.5 | 42.6 | 14.7 | 32.4 | 22.1 | 25 |
| 2 | 6.0 | 17.6 | 11.8 | 29.4 | 1.5 | 26.5 | 22.1 |
| 3 | 0 | 41.2 | 0 | 45.6 | 1.5 | 38.2 | 32.4 |
| 4 | 0 | 4.4 | 0 | 1.5 | 0 | 8.8 | 4.4 |

*Agreement with stereotypical statements regarding Tourette syndrome in percent (%). ‘Strongly disagree’ = 0, ‘disagree’ =1, ‘neither agree nor disagree’= 2, ‘agree’ = 3, ‘strongly agree’ = 4.*

**A4.**

|  | Tourette Syndrome | Schizophrenia |
| --- | --- | --- |
| 0 - Never | 7.2 | 2.9 |
| 1 - Not often | 66.7 | 62.3 |
| 2 - Often | 20.3 | 26.1 |
| 4 - Quite Often | 5.8 | 8.7 |

*Ratings of the item ‘How often have you come across media portrayals of individuals with Schizophrenia/Tourette Syndrome, such as characters in films and TV programs, social media posts, etc?’ in percent (%).*

**A5.**

|  | 1 | 2 | 3 |
| --- | --- | --- | --- |
| 1 - TS Attitude Score |  |  |  |
| 2 - Daily Social Media Hours | .00 (.980) [>.999] |  |  |
| 3 - Seen TS Media Portrayals | -.19 (.128) [.768] | .02 (.882) [>.999] |  |
| 4 - Media influenced my Perception | .06 (.644) [>.999] | .08 (.514) [>.999] | .18 (.141) [.768] |

*Results of Pearson Correlations with r, p-values in brackets () and Bonferroni-Holm adjusted p-values in squared brackets [], between the variables 1 - ‘Tourette Syndrome (TS) Attitude Score’ 2 - ‘daily hours spent on social media’, 3 - ‘Frequency of seen TS media portrayals’, and 4 - ‘Belief that the media influenced one’s view about TS’.*

**A6.**

|  | 1 | 2 | 3 |
| --- | --- | --- | --- |
| 1 - SCZ Negative Attitude Score |  |  |  |
| 2 - Daily social media hours | .03 (.783) [.783] |  |  |
| 3 - Seen SCZ Media Portrayals | -.19 (.130) [.448] | .26 (.028) [.140] |  |
| 4 - Media - Opinion | .28 (.020) [.120] | .19 (.121) [.448] | .19 (.112) [.448] |

*Results of Pearson correlations with r, p-values () in brackets and Bonferroni-Holm adjusted p-values in squared brackets []* *between the variables 1 - ‘Schizophrenia (SCZ) Attitude Score’ 2 - ‘daily hours spent on social media’, 3 - ‘Frequency of seen SCZ media portrayals’, and 4 - ‘Belief that the media influenced one’s view about SCZ.*
